# “Optima attempts to objectively and pragmatically assist countries meet their targets most efficiently and effectively”

**DOI:** 10.1002/jia2.25190

**Published:** 2018-10-14

**Authors:** David P Wilson, Marelize Gorgens, David J Wilson, the Optima HIV study team

**Affiliations:** ^1^ Burnet Institute Melbourne Vic. Australia; ^2^ World Bank Washington DC USA

**Keywords:** cost‐effectiveness, modelling, efficiency, resource allocation

Dear Editor,

The global HIV disease burden remains significant and the targets of 90‐90‐90 are important to keep the focus on eliminating HIV as a public health threat. But we acknowledge that we have “miles to go” before the 90‐90‐90 targets are reached 1. Granich, Gupta and Williams 2 may not have been aware of how Optima has been used to support these global targets nor of the practical policy questions for which Optima has been used and may have misinterpreted some of our results. The Optima HIV model was created in an attempt to assist decision‐making around prioritization of HIV interventions when resource constraints mean that prioritization is essential. While countries are generally concerned with the question of how to reach the global targets of 90‐90‐90 and 95‐95‐95, they also want to know how they can use their existing resources in the most efficient way, while mobilizing additional funds to expand service coverage. The Optima HIV model allows countries to define their own policy questions, and collate and validate the data required to answer these questions (as noted in Table 1 in our article). Importantly, this means that the analyses investigate questions considered important by country teams, including what it would take to achieve 90‐90‐90, and other questions of interest to them. In our paper, we summarized the results of 23 studies in which the central question of concern for countries was how to maximize the health outcomes of their HIV responses assuming that no additional funds are immediately available 3. Focusing on improving programmatic efficiency in the present moment does not preclude focusing on longer‐term targets in the future. Indeed, we have shown long‐term support of these laudable targets: not only was Optima HIV used in the initial modelling efforts around the release of the first UNAIDS launch of the 90‐90‐90 strategy 4, but we also have since continued to promote this strategy in other academic publications relevant to our local setting and internationally (e.g. 5, 6, 7, 8, 9). Whenever country teams work with the Optima HIV model, our recommendation has been to explicitly model 90‐90‐90 targets and many countries chose to do so in the original studies using the model. Our research using Optima HIV has also demonstrated that scaling‐up treatment is typically of highest priority for most countries 10, and this was also a key result in the studies summarized in this article.

While we collectively aspire to full coverage of all services for all people in need, especially including testing and treatment, the unfortunate reality is that complete implementation of 90‐90‐90/95‐95‐95 may not be feasible to implement in some contexts. The letter and modelling studies by Granich, Gupta and Williams advocate for testing everyone in high‐prevalence settings once or twice a year and providing immediate treatment 2. While we agree with the personal and population benefits if this could be achieved, only focusing on this strategy at the expense of a comprehensive response targeting the needs of people in each context and addressing the country's specific strategic targets may cause more overall harm by diverting resources from the most cost‐effective approaches to achieve country targets. Countries weigh their HIV investments against their specific HIV objectives and other health, social sector, development and economic investments in an attempt to achieve universal health coverage. We need to consider the marginal value of all investments. Other diseases such as TB and HCV now have 90‐90‐90 targets, which receive much less investment than HIV.

In achieving countries’ goals of eradicating HIV, improvements in allocative efficiency, technical efficiency, production efficiency and social efficiency are needed. These need to work together in a synergistic way. In response to the authors’ comments about technical efficiency, we would simply point out that technical efficiency gains were explicitly discussed in the article and in several of our related papers 11. The same applies to the comments about the preventative benefits of treatment, which the authors erroneously claim are omitted from the model despite information to the contrary clearly shown in the model documentation.

Granich, Gupta and Williams claim that our model results indicate an “allocation away from treatment,” but our results clearly indicate the opposite. To suggest that the model suggests unethical decision around removing people from treatment is simply not true: the model has built‐in constraints to ensure that no one put on treatment, would be taken off treatment. The average ART coverage across all 23 countries in the year that the studies were conducted (which ranged from 2011 to 2014) was only 30%, and our analyses recommended *immediately* increasing this to 42% – already an ambitious target, and not at all in conflict with the idea of looking at a more significant scale‐up over a longer time horizon, which we typically simulated with our model. We would not recommend that this be used as a global benchmark, as the allocative efficiency of every country's HIV response will differ based on a multitude of characteristics. We believe Granich, Gupta and Williams miss the central concern of our studies, which was to address the question of what could be done immediately with existing funding, as opposed to what could be done over longer time horizons with an increased budget.

The authors’ main critique of Optima seems to be that it does not solely proclaim the global 90‐90‐90 by 2020/95‐95‐95 by 2030 strategy at the exclusion of any other strategy, whether prioritized in a country national strategic plan or not. The authors cite their own publications in which they make very optimistic assumptions around coverage and effectiveness of ART, the future reduced costs of delivering ART and reductions in risk behaviour for people on ART. The extent of their assumptions have limited scientific basis but are consistent with the promotion of the singular 90‐90‐90/95‐95‐95 strategy. We are very willing to engage with critiques of the model on scientific grounds; however, the critiques outlined in this letter seem to originate from a political agenda more than a scientific one.

## Competing interests

The authors declare that they have no competing interests.

## Authors’ contribution

DPW, MG and DJW equally contributed to this response, supported with edits from the Optima HIV study team.
